# Does suffering suffice? An experimental assessment of desert retributivism

**DOI:** 10.1371/journal.pone.0230304

**Published:** 2020-04-20

**Authors:** Paul C. Bauer, Andrei Poama

**Affiliations:** 1 Mannheim Centre for European Social Research (MZES), University of Mannheim, Mannheim, Germany; 2 Leiden University, The Hague, Netherlands; Shandong University of Science and Technology, CHINA

## Abstract

Michael S. Moore is among the most prominent normative theorists to argue that retributive justice, understood as the deserved suffering of offenders, justifies punishment. Moore claims that the principle of retributive justice is pervasively supported by our judgments of justice and sufficient to ground punishment. We offer an experimental assessment of these two claims, (1) the pervasiveness claim, according to which people are widely prone to endorse retributive judgments, and (2) the sufficiency claim, according to which no non-retributive principle is necessary for justifying punishment. We test these two claims in a survey and a related survey experiment in which we present participants (N = ~900) with the stylized description of a criminal case. Our results seem to invalidate claim (1) and provide mixed results concerning claim (2). We conclude that retributive justice theories which advance either of these two claims need to reassess their evidential support.

## Introduction

Those who think that criminal punishment ought to serve a retributive function form a big philosophical family whose distinctive identity is not easy to pin down. For instance, [1] counts nine varieties of retributivism, to which [2] adds four more. Retributive conceptions can cover a wide range of rationales, such as deserved penal suffering, a concern for proportionality in the enforcement of criminal sanctions, the imposition of punishment as part of a fair institutional scheme or a concern for expressing the validity of those values putatively violated through criminal offenses.

The present study looks at one specific, and arguably the most philosophically sophisticated version of retributivism, namely, Michael S. Moore’s (1997) retributive justice account of criminal punishment ([3], see [4–9] for other positions). Our purpose is to examine whether one of the central claims on which Moore rests his retributive justice account of criminal punishment—the claim that making guilty criminal wrongdoers suffer is sufficient for realizing justice through punishment—is empirically corroborated by ordinary judgments of justice in a survey experiment. We ask: *Does suffering suffice?* The aim is to assess whether, on reflection, people tend to judge that the penal suffering of criminal offenders is enough for penal justice to be done. Additionally, we aim to assess whether retributive beliefs as construed by Moore—i.e., beliefs that deserved suffering is sufficient for punishing—are widely supported or not.

The reason for focusing on Moore’s version of retributivism is threefold. First, Moore’s version of retributivism is *positively* retributivist, meaning that he takes deserved suffering to generate an obligation to punish, as distinct from merely rendering punishment permissible, which is an idea defended by negative or weak retributivists [10]. Whether the idea of deserved suffering is positively supported will have implications for negative or weak forms of retributivism, where punishment should not be imposed above levels that are deserved. This is because if something does not count as an obligation, it is unlikely that it can function as a side-constraint. Second, Moore’s theory is systematic in a way that other forms of retributivism are not. Because of this feature, assessing the soundness of parts of the theory will have implications about other parts of the theory and, more broadly, might matter for how we think about just punishment. Third, Moore’s theory lends itself to empirical assessment (given its meta-ethical, epistemic and methodological commitments, i.e., moral realism and reflective equilibrium), which is an advantage for any experimental test (empirical assessment here should be understood in terms of actual normative support, not in terms of predictive or explanatory power). Roughly put, moral realism refers to the theory that moral judgments and intuitions are about real (moral) states of affairs/facts which can be observed by any competent moral agent under normal conditions. The epistemological claim here is that moral states of affairs can be known via intuitions or judgments, and that these judgments can be widely shared. This renders moral realism sensitive to research about what most people actually intuit or judge as morally right or wrong about specific states of affairs (in our case, punishment). Experimental findings, then, are something moral realists have epistemic reasons to care about. Reflective equilibrium refers to the moral reasoning method whereby moral theories are decided by examining whether particular judgments about particular cases cohere with each other and with other general beliefs (some of which are principles) about a wider range of cases. Experimental surveys can be considered as tools for generating similar particular cases (via experimental conditions) and for examining the corresponding judgments in order to come up with a theory that coherently accounts for them.

Our paper brings a threefold contribution. First, it weighs in on the normative literature on punishment by providing an empirical measure of the extent to which retributive judgments are reflectively supported by lay persons. This answers some calls which have been recently made by normative theorists of punishment about the importance of assessing the generality, strength and stability of retributive judgments and intuitions [11]. Our paper focuses on questions of generality and strength.

Second, we bring experimental techniques to bear on questions of legal punishment, a topic which, with some exceptions has not been the direct concern of experimental philosophers up until now [12–14]. Few studies, focus on punishment as a general moral phenomenon, and not on judgments about legal punishment in particular [15–17].

Third, we aim to contribute to the experimental philosophy literature not only substantively—namely, by drawing attention to questions of punishment and retribution—but also methodologically. At this level, following previous authors [18, 19] we attempt to show that open-ended questions can add an extremely informative dimension to philosophical experiments. This is because open-ended questions allow for testing whether respondents hold certain ideas without priming them to answer in pre-defined ways. We think that researchers can use this strategy to enhance their experimental designs with more qualitative knowledge, a move that has been called for lately [20].

This paper proceeds as follows. In Section 2 we outline Moore’s retributive theory and the hypotheses we formulate based on this theory. Section 3 presents our experimental design, the data and the methods applied. In Section 4, we provide a summary of our results. Section 5 offers a discussion and philosophical interpretation of these results. Section 6 provides a conclusion.

## Theory and hypotheses

As Moore understands it, retributive justice offers a general formulation of our considered judgments and intuitions about cases concerning the evaluation and treatment of serious criminal wrongs. Moore’s account of retributive justice relies on a non-foundationalist methodology. This is to say that the principle of retributive justice is not deduced from a higher-order principle, but is instead derived via abductive reasoning from particular judgments, views and intuitions about specific cases of criminal wrongs. The main cases Moore considers are mostly cases of *mala in se*, which are wrongs that would count as such even in the absence of their legal criminalization. Typically, these are cases of murder, rape or other similar violent wrongs against persons. Were we to reflect seriously about what should be done in response to such wrongs, Moore argues that most of us would consider that the suffering of wrongdoers is what justice requires. For example, imagine you were a man who killed his girlfriend because, while sleeping, she released herself from your embrace. Moore holds that, on reflection, each of us would rightly ‘feel guilty unto death’ about such a murder and that, for each of us, these feelings of guilt would properly lead to the following judgment: ‘I could not imagine any suffering that could be imposed upon me that would be unfair because it exceeded what I deserved’ [3]. Moore refers to this so-called Richard Herrin case to elicit retributive judgments. For a more detailed account of this case, see, for example, [21].

After considering other similar cases of wrongdoing—for instance, real cases of rape or the fictitious case of Dostoyevsky’s nobleman who lets his dogs tear apart a child in front of his mother [3]—Moore concludes that ‘the properties of wrongdoing and culpability cause most of us to believe that, when we have culpably done wrong, such acts are evil and that we are guilty. Given the duty to suffer brought into existence by our culpable wrongdoing, our belief that we are guilty includes a belief that we must suffer (i.e., be punished)’ [3]. Moore takes these cases to be ‘good evidence for the truth of the retributive principle that culpable wrongdoers must be made to suffer’ [3]. Because we have primary duties ‘not to do the sort of acts that malum in se criminal statutes prohibit,’ we have acquired ‘secondary duties to allow ourselves to be made to suffer if we have violated these primary duties’ [3].

Thus understood, the principle of retributive justice is a principle of deserved suffering that is, duty owed suffering. In short, punishment is justified when it brings about deserved suffering. Moore’s case for retributive justice relies on the following two claims. The first claim is that retributive judgments will be pervasively shared by people as the adequate way of thinking about the just response to serious criminal wrongdoing. We call this the pervasiveness claim:

*Pervasiveness Claim: on reflection, people pervasively believe that deserved suffering is a sufficient reason for punishing guilty wrongdoers*.

We operationalize the pervasiveness claim in terms of the following hypothesis:

A plurality (the largest group) of people thinks that the most important aim of punishment is to make guilty offenders suffer. (H1)

Note that our interpretation of the concept of ‘pervasively shared judgment’ is relatively permissive. We do not require that a majority (or supermajority) of people hold or support the same type of judgment (in this case, a retributive one) for that judgment to be considered as pervasively shared. This permissive interpretation of ‘pervasiveness’ is motivated by the fact that people will often find themselves disagreeing on complex normative questions (like the norms of justice that matter for punishment), such that the likelihood of a majority viewpoint on these questions remains realistically low. Consequently, plurality, understood as the greatest number of views partaking in the same viewpoint as compared to other viewpoints, seems to be the appropriate way to think about pervasiveness in the context of normative disagreement. We test H1 with three measures, a survey question asking participants an open question about the possible aims for punishment, a survey question letting them rank seven general justifications of punishment, and an explicit retributivist question.

Additionally, we examine Moore’s claim that the principle of retributive justice is a sufficient justificatory condition for criminal punishment to be just, and, more generally, for justifying the existence of criminal law as a distinctive area of law [3]. This is to say that, if criminal punishment brings about deserved suffering, no other normatively condition needs to be met in order for punishment to be justified and, in particular, for it to be just. For justice to be done through punishment, it is sufficient for criminal wrongdoers to be made to suffer. More simply, penal justice is done whenever criminal wrongdoers suffer by means of punishment. That penal justice is retributive should be understood in terms of criminal wrongdoers’ deserved suffering being a sufficient reason for rendering the practice of punishment just. In what follows, we will refer to the claim that deserved suffering is sufficient for justice to be done through punishment as the sufficiency claim. Moore’s defense of the sufficiency claim can be summarized as follows:

*Sufficiency Claim: suffering alone is sufficient to realize justice, i.e., no other morally relevant condition is necessary to realize justice (for example, deterrence or rehabilitation, compensation or reparation)*.

Moore comes close to this concise formulation when he argues that, if wrongdoers are made to suffer, no other normatively relevant condition needs to be satisfied for punishment to be just. In Moore’s terms, ‘within the set of conditions constituting intelligible reasons to punish, the retributivist asserts, desert is sufficient, i.e., no other of these conditions is necessary’ [3]. We operationalize Moore’s sufficiency claim in terms of two hypotheses that *jointly* allow us to assess whether people reflectively endorse the claim. This is to say that the sufficiency claim can be empirically vindicated only if *both* of the above hypotheses are corroborated. This is because the two hypotheses capture two constitutive dimensions that any sufficiency relation is supposed to satisfy. The first dimension (captured by H2) is the positive valence of the relation between the thing that counts as a sufficiency condition and the thing for which the first one is a sufficient condition for. The second dimension (captured by H3) is the non-necessity of alternate conditions. This is to say that, if a thing is a sufficient condition for something else, then no other thing is needed for that something to obtain.

The first (positive valence) dimension of the sufficiency claim is operationalized as follows:

Judgments of justice are positively affected by perceptions of the offender’s suffering. (H2)

H2 offers an indirect and testable formulation of the sufficiency claim. Saying that a guilty offender’s deserved suffering is sufficient for realizing penal justice commits Moore to the proposition that, when (and insofar as) offenders suffer, people will perceive that justice has been done. More generally, holding that X is sufficient for Y entails that X is positively correlated with Y and that, whenever X occurs, Y will also occur, at least to a certain (satisfactory) extent. Translated in terms of positions on a scale of perceived justice, sufficiency entails a positive relation—namely, a positive correlation—between the sufficiency condition (suffering) and the phenomenon for which that condition is deemed sufficient (perceived justice). Consequently, if suffering is a sufficient condition for justice, suffering cannot be negatively correlated with perceived justice. The fact of suffering occurring should not, by itself, lead to lower values of perceived justice. We test H2 on the basis of evidence provided by people’s elicited justice judgments about the punishment of a criminal rape case (see next section). Our aim is to see whether people’s judgments about the justice of punishment in this paradigmatic (*malum in se*) case corroborate the claim that suffering alone is a sufficient condition for punishment to be just.

Another way to assess the claim that retributive suffering is a sufficient reason for punishing (sufficiency claim), is to see whether other standard reasons for punishing—such as prevention or rehabilitation—are pervasively held by individuals or do influence them in their judgments of justice. This is to say there might be other reasons for punishing, and that these reasons might be necessary in order for punishment to be deemed just. In what follows, we concentrate on such another reason that we will refer to as the moral change of the offender. Moral change refers to the offender’s willingness to repair or otherwise make up for his crime. We expect that an offender’s moral change in relation to her crime may be considered as an even more important or at least equally important principle of justice. This captures the second (non-necessity of alternate conditions) dimension of sufficiency relations. We operationalize this second dimension as follows:

Judgments of justice are positively affected by an offender’s display of moral change. (H3)

The reasons for concentrating on moral change are both normative and empirical. Normatively, we think that there are good arguments for thinking that suffering is, at best, a by-product of the process whereby justice is done in relation to crime, and not its aim. For example, Victor Tadros notes that we should see suffering as a side-effect of a conflict of practical reasoning between the wrongdoer’s ‘judgments at the time of acting’—that is, at the time of committing the crime—and his ‘judgments now’—that is, at the moment when he recognizes that the crime is a wrong [22]. Tadros notes that ‘it is hard to believe that a person who recognizes that what she has done is wrong feels guilty because she thinks that she deserves to suffer’ [22], and argues that the more plausible explanation goes the other way around. To wit, the person might suffer because she thinks she has done wrong. Similarly, and somewhat more colorfully, Martha Nussbaum argues that the idea that suffering by itself brings about justice is ultimately a form of ‘magical thinking’ [23]. More generally, Parfit suggests that the belief that suffering *in itself* is valuable could be debunked *qua* normative belief if shown to be a by-product of human evolution [24]. Empirically, there are some experimental findings showing that people tend to consider that punishment is just, not because it leads to the wrongdoer’s suffering per se, but because the wrongdoer acknowledges the wrong that (s)he has committed and is willing to make up for it. For example, Funk has shown that, for participants who decided to impose a sanction on (confederate) transgressors in a lab experiment, ‘the perception of suffering did not correlate with justice-related satisfaction overall’ [25]. However, most of the studies showing that suffering as such does not provide a sufficient reason for justice pertain to situations whose structure and content do not match real-life cases of punishing actual crimes. Given that we are focusing on a realistic formulation of a real-life case, one of the strengths of our experimental study resides in its comparatively higher ecological validity.

## Experimental design, data and methods

We test our hypotheses relying on data from a survey and a corresponding survey experiment. In January 2018 we collected a sample of N = 940 survey respondents online on the plattform prolific.ac. The data was collected and analyzed anonymously. The study was approved of by the Ethical Commission of the University of Mannheim (Nr. EK Mannheim 2/2018). On average participants spent around 5.3 minutes to answer the survey and each participant was paid 0.42 GBP (~0.48 EUR) on average, which amounts to an average hourly wage of 5.04 GBP (~5.73 EUR). Following a fair payment policy, Prolific.ac requires that participants on the platform are paid at least the minimum hourly wage.

**Fig 1:**
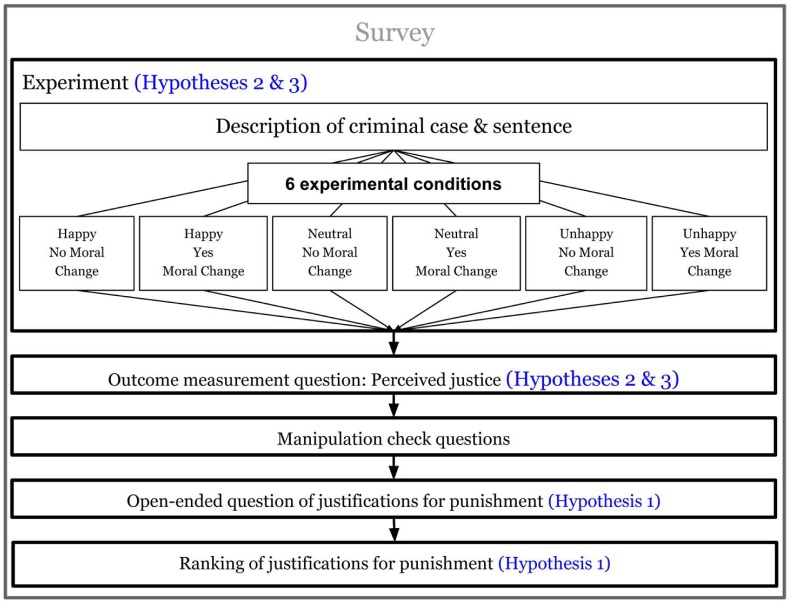
Setup of the survey.

The setup of our survey and experiment is depicted in Fig 1. Participants were first presented with a survey experiment about a stylized criminal rape case [3] that assessed Moore’s sufficiency claim (testing of H2 and H3). Subsequently, participants were presented with survey questions asking for the most important aims of punishing offenders in both an open-ended and a ranking form, as well as with an explicit retributivist question (see below). We use these three questions to assess Moore’s pervasiveness claim (testing of H1). Administering the survey about the general justifications of punishment after the experimental survey of the stylized case allowed us to avoid priming effects. The study contained a total of 7 questions and we obtained additional socio-demographic data from prolific.ac. Hypothesis 1—the pervasiveness claim—was tested through three questions.

1. *What do you think should be the most important aim of punishing offenders? Please provide a short answer*. The advantage of this open question is that we avoid priming respondents by NOT providing them with a predetermined set of choices or a scale. Subsequently, their answers are categorized relying on crowdcoding. Thereby, each response is classified by 4 raters. The setup of the rating task (number of raters, average rating times, etc.) and the design of the rating task are described in the S1 Appendix.
  We do not take open-ended answers to be perfect measures for any of our two hypotheses. Rather, we see them as complementary ones, insofar as they can shed new light on the reasons ordinary people have for their answers. Open-ended talk about reasons is not uninformative. Using open-ended questions is not uncommon in experimental research, both online and in-lab [18, 19, 26, 27], and has been presented as a strategy for tapping into the views that participants deeply care about and which are otherwise blocked by a closed-answer design [20]. For instance, Nadelhoffer (2013) stresses that open-ended questions might offer insights into participants’ emotions about their behavior [13]. This is arguably relevant for a theory (like Moore’s) that appeals to the normative value of reactive attitudes such as guilt and indignation. Open-ended questions might thus offer experimental philosophers a (partial) reply to critics who worry about the ability of experimental surveys to capture the detailed or otherwise ignored reasons that people have for holding a particular normative position [28]. Finally, and more generally, recent survey research suggests that open-ended answers are not necessarily less accurate than closed-ended ones [29].
2. *What do you think should be the most important aims when punishing offenders? Please rank all the aims of punishment on the list below according to their importance from top = most important (Number 1) to bottom = least important (Number 5). All your answers must be different and you must rank in order. Please number each box in order of preference from 1 to 5: (1) To prevent future crime (2); To give offenders what they deserve…; (3) To reform and rehabilitate offenders…; (4) To bring offenders to make amends to the victim…; (5) To show society’s disapproval of the crime*. The aims of punishment that we selected are synthetically based on public opinion surveys used in criminological research about sentencing aims and principles [30]. See [31] for a discussion about the normative relevance of sentencing aims surveys.
3. *Do people who commit crimes deserve to suffer as a result of punishment, even if their suffering will not produce any positive benefits for the offender or society, like rehabilitation or the prevention of future crime? Please choose a value from 0 to 10 below, where 0 means ‘People who commit crimes do not deserve to suffer if there are no other benefits’ and 10 means ‘People who commit crimes deserve to suffer even if there are no other benefits’*“. Following [13], we call this the explicit retributivist question.

To our knowledge, this is the best available way to measure explicit retributivism. Another option would have been to use questions, where participants are given two brief summary explanations of retributivism and consequentialism, and are then asked the extent to which they agree with the two following statements [32]:”Please indicate how important you feel each motivation should be to criminal punishment: (1) I feel that retributivism should be an important motivation in criminal punishment. (2) I feel that consequentialism should be an important motivation in criminal punishment“. We find these questions inadequate for measuring distinctly retributive attitudes. First, the statements are ambiguous between moral justifications and psychological motivations, so that it is not clear that they capture participants’ views about what justifies punishment morally, and not their views about what they think motivates humans. Second, rating consequentialism and retributivism in terms of their relative importance does not tell us much about the sufficiency claim, if we understand it as a claim about non-retributivist aims not being necessary for justifying punishment. One way to rephrase the statement here would have been the following:”supposing that the consequentialist justification of punishment is not true, to what extent do you agree with the statement that retributivism justifies punishment?” But this seems contrived. Third, the way in which retributivism is explained does not exclude the idea that deserved suffering might be instrumental to a non-retributivist value. Participants might have understood consequentialism to combine two aims: deserved suffering and a more general aim (social utility, rehabilitation, etc.) to which deserved suffering might be conducive. This can be corrected, but it makes the measure of retributivism used in [32] problematic.

The hypothesis that a *plurality of people consider that the just response to serious criminal wrongdoing consists in making guilty offenders suffer for their crimes*, would be undermined if, first, only few people choose “to give offenders what they deserve” in a plurality of cases and, second, if a plurality of people provide low scores on the explicit retributivist question. There are reasons to believe that support for this second aim—give offenders what they deserve—will translate in lower levels for deserved suffering. This is because the adequate account of desert claims is pluralistic, which implies that deserved treatments in cases of criminal wrongdoing are not equivalent to deserved suffering, but might include other forms of deserved treatment as well, such as deserved redress costs [33].

Our experimental test of *Hypothesis 2 and 3* focuses on a stylized version of a real criminal case that is repeatedly used by Moore to elicit the particular retributive judgments on the basis of which he infers his principle of retributive justice. The case is State v. Chaney (1970), and Moore summarizes it as follows:

In Chaney, the defendant (Timothy Chaney) was tried and convicted for two counts of forcible rape, and one count of robbery. The defendant and a companion had picked up the prosecutrix at a downtown location in Anchorage. After driving the victim around in their car, the defendant and his companion beat her and forcibly raped her four times, also forcing her to perform an act of fellatio with the defendant’s companion. During this same period of time, the victim’s money was removed from her purse, and she only then was allowed to leave the vehicle after dire threats of reprisals if she attempted to report the incident to the police [3].

Moore then modifies this case into a thought experiment that putatively elicits retributive judgments. He writes:

The thought experiment that such a case begins to pose us is as follows: Imagine in such a case that the defendant after the rape but before sentencing has got into an accident so his sexual and aggressive desires are dampened to such an extent that he presents no further danger of violence against women; if money was also one of his problems, suppose further that he has inherited a great deal of money, so that he no longer needs to rob. Suppose, because of both of these facts, we are reasonably certain that he does not present a danger of either forcible assault, rape, or robbery or related crimes in the future. Since Chaney is (by hypothesis) not dangerous, he does not need to be incapacitated, specially deterred, or reformed. Suppose further that we could successfully pretend to punish Chaney, instead of actually punishing him, and that no one will find out [3].

According to Moore, this modification of the case allows us to see whether the defendant suffering from punishment is still a sufficient reason to punish him once it is clear that, given the content of case, no other clear consequentialist good (such as prevention or rehabilitation) will come out of his punishment. Moore thinks that most of us would still consider punishment to be the just thing to do in such a (thought experimentally modified) case and that the reason for this is adequately provided by the duty the offender has to allow himself to be made to suffer via punishment. He takes such a judgment about the justice of (consequentially ineffective or irrelevant) punishment to provide evidence for the truth of the principle of retributive justice.

To test Hypotheses 2 and 3 we conduct a survey experiment. In our design, we have 2 treatment variables that split the participant sample into 6 experimental conditions: Suffering, i.e., whether a sentence makes the offender suffer with three categories, “happy”, “neutral” and “unhappy”; MoralChange, i.e., whether a sentence makes the offender modify his moral stance with two categories, “no moral change” and “yes moral change”. These variables are formulated as vignettes in our survey experiment. See Fig 1 for the setup.

The introduction of the happy category is meant to ensure a more fine-grained assessment of the offender’s suffering on judgments of justice [25]. We long discussed about how “suffering” should be operationalized. We finally settled for “happy”/“not happy”/“neither happy nor unhappy” for three reasons. First, choosing stronger terms (for instance: “distressed”, “miserable”, “depressed” etc.) might have elicited sympathy reactions in our participants, which would not have been charitable for measuring desert retributivists’ conception of suffering. Choosing terms like “remorseful”, “repentant” or “apologetic”, on the other hand, might have led participants to view suffering as merely instrumental to a different value, like moral change, and could have therefore failed to capture the desert retributivists’ claim that suffering is intrinsically valuable. Second, choosing “happy” and its negations allowed us to cleanly compare the different experimental conditions in a way that the use of partial antonyms (for instance, “distressed” vs. “content”) couldn’t have. Semantically, the terms we chose are on a par, which is methodologically fortunate. Third, our option in favor of a hedonic operationalization of “suffering” is ultimately a normative choice. In understanding suffering as absent happiness, we draw on [34], who contrasts suffering with happiness, and argues that the psychological definition of suffering (as “a state of feeling bad overall, or disagreeable overall feeling”, 14) is one that would be shared by most competent users of the term. If definitions are reports on linguistic use and Mayerfeld is right about the ordinary use of the term “suffering”, our hedonic operationalization of suffering is arguably on the right track. This does not mean that other operationalizations of suffering should not be used in future experimental studies (“feeling bad” might be another option).

As visualized in Fig 1, participants first get a description of a criminal case and a sentence:

On February 7, 2014, David Chadwick picked up Julia Arneson in his car, while she was waiting at a crossroad. After driving around for a while, David stopped in an open field, where he beat and raped Julia. David then drove back to the crossroad and left Julia there. At the time of his trial, it was disclosed that David had no prior criminal record. Shortly after committing his crime, David suffered a head injury in an accident. Medical experts confirmed that the risk that David commits a similar offence in the future is, as a consequence, practically nonexistent. Moreover, criminological studies show that other individuals similar to David will in no way be affected by the sentence he receives. Following his trial, David Chadwick was sentenced to 10 years imprisonment without the possibility of being released earlier.

This description is followed by six conditions/treatments to which participants are randomly assigned and which vary the levels of our two variables:

- Treatment 1 (happy/nomoralch): Because of favourable confinement conditions, David spent most of his days in prison feeling very happy. While in prison, he was allowed to make amends for his crime. Despite of this, he did not write Julia any apology letter nor asked to work from prison for an organization helping women who are victims of sexual abuse.
- Treatment 2 (happy/yesmoralch): Because of favourable confinement conditions, David spent most of his days in prison feeling very happy. While in prison, he was allowed to make amends for his crime. Due to this, he wrote Julia several apology letters and asked to work from prison for an organization helping women who are victims of sexual abuse.
- Treatment 3 (neutral/nomoralch): Because of average confinement conditions, David spent most of his days in prison feeling neither happy nor unhappy. While in prison, he was allowed to make amends for his crime. Despite of this, he did not write Julia any apology letter nor asked to work from prison for an organization helping women who are victims of sexual abuse.
- Treatment 4 (neutral/yesmoralch): Because of average confinement conditions, David spent most of his days in prison feeling neither happy nor unhappy. While in prison, he was allowed to make amends for his crime. Due to this, he wrote Julia several apology letters and asked to work from prison for an organization helping women who are victims of sexual abuse.
- Treatment 5 (unhappy/nomoralch): Because of strict confinement conditions, David spent most of his days in prison feeling very unhappy. While in prison, he was allowed to make amends for his crime. Despite of this, he did not write Julia any apology letter nor asked to work from prison for an organization helping women who are victims of sexual abuse.
- Treatment 6 (unhappy/yesmoralch): Because of strict confinement conditions, David spent most of his days in prison feeling very unhappy. While in prison, he was allowed to make amends for his crime. Due to this, he wrote Julia several apology letters and asked to work from prison for an organization helping women who are victims of sexual abuse.

The outcome variable is judgments of justice, namely the extent to which a respondent judges the sentence/punishment to be just, i.e., to satisfy justice. It is measured as follows: “Below you find a scale from 0 to 10 where 0 means ‘justice has not been done at all’ and 10 means ‘justice has been fully done’. Do you think that justice has been done in David Chadwick’s case?”. We also probed respondents directly after this question by asking ‘You chose a value of X on the justice scale? Could you explain in a few words why you picked this value?’. However, in the present paper we refrain from analyzing this data.

**Fig 2:**
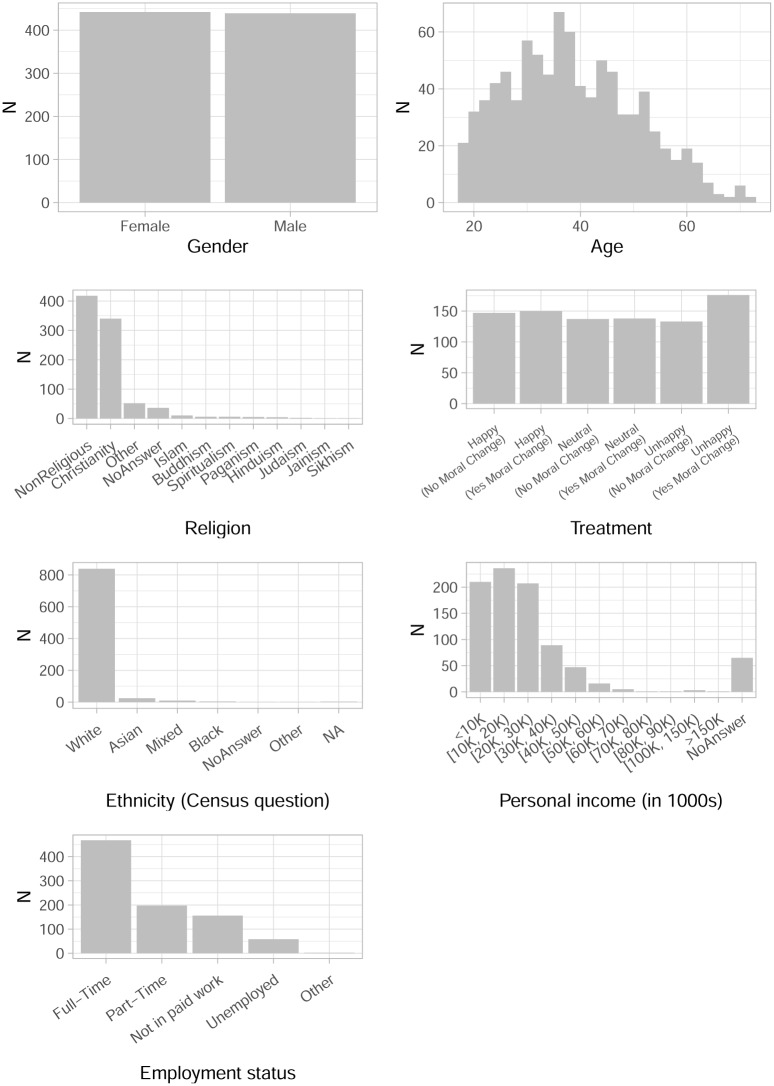
Covariate distributions in the sample.

As illustrated by Fig 2 the sample comprises roughly the same number of women and men and a wide distribution of ages starting at 18. The most common categories of religion are non-religious and Christianity, most participants work full-time, are white and earn between 10000 and 19999 British pounds. Balance across treatment groups is satisfactory (see S1 Appendix).

## Results

### Hypothesis 1: Pervasiveness of retributive views

The first of Moore’s claims concerns the pervasiveness of retributive views of justice. From his argument we derived H1, namely that retributive considerations are supported by a plurality—the largest group—of people. We test H1 relying on three survey questions: An open-ended question (1), a ranking question querying aims of punishment (2) and a rating scale for deserved suffering (3).

Table 1 displays the open-ended responses after they have been classified according to whether they mentioned particular aims of punishment. For instance, row 1 displays the share of participants whose open-ended response mentioned “suffering” as an aim of punishment. The data obtained through the open-ended question provides evidence that retributive considerations are not supported by a plurality of people. When asked in an open-ended fashion most people will mention awareness (the aim of punishment is to make the offender aware of what he/she did), rehabilitation or deterrence as aim of punishment. Far fewer consider suffering as an aim.

**Table 1:** Share of open-ended answers that mention particular aims.

	% responses	N responses
Mention aim of suffering	12	108
Mention aim of deterrence	35	304
Mention aim of reintegration	2	18
Mention aim of rehabilitation	32	280
Mention aim of amends	5	40
Mention aim of vengeance	4	33
Mention aim of awareness	23	204

**Fig 3:**
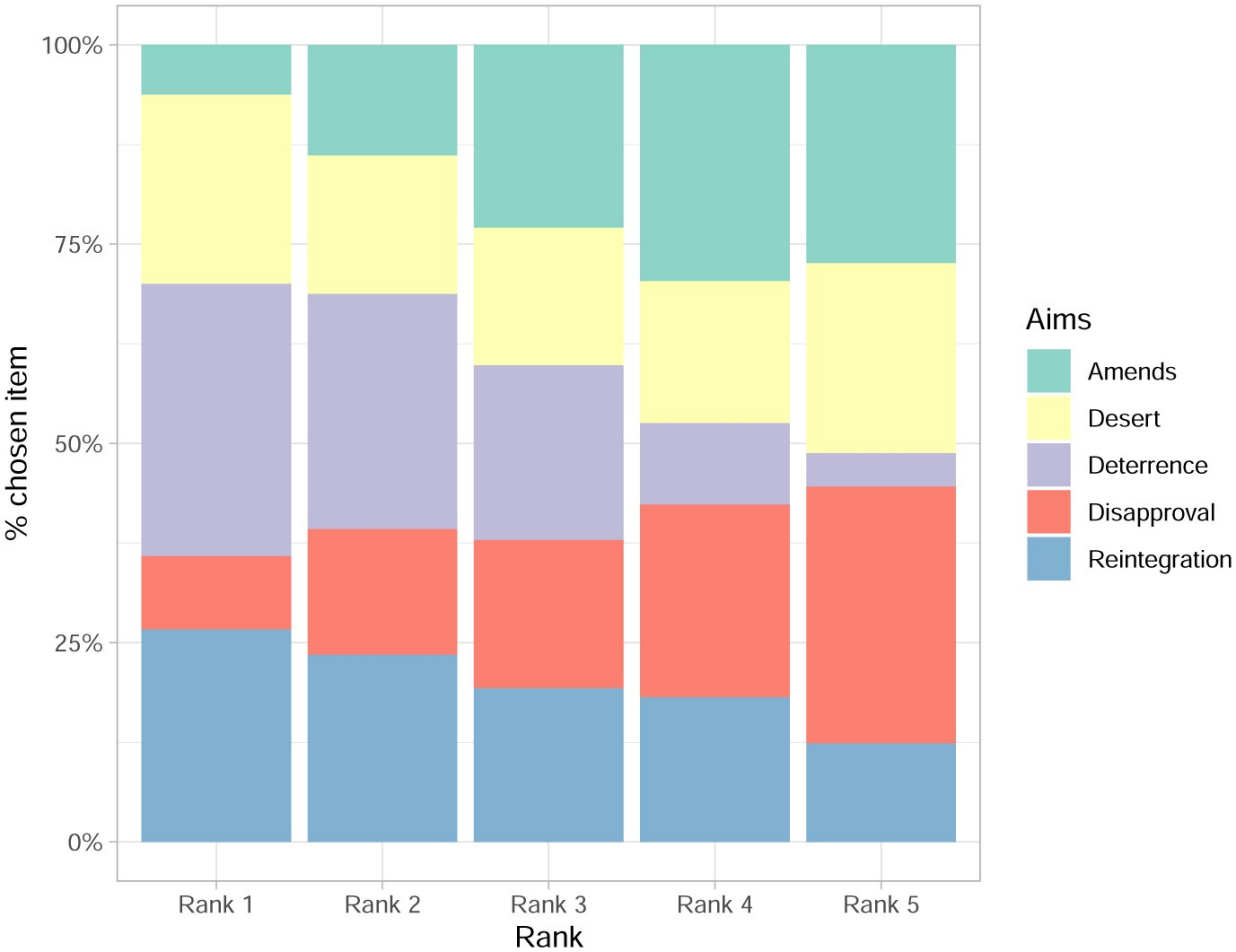
Ranking aims of punishment.

Fig 3 visualizes the results of the ranking question that provides respondents with a pre-defined choice set. The corresponding numbers can be found in Table 2. Fig 2 shows that the justification items ranked at position 1 most often are deterrence 34%, reintegration 27% and suffering 24%. We included respondents who decided not to rank any justification items in those calculations. In other words, when compared to other reasons for punishment, desert is only ranked first by the third largest share of people. Besides, a relatively low percentage ranks it first at all, namely 24% percent. The desert option in the ranking question arguably overestimates the extent to which people take deserved suffering to be the most important aim of punishment. This is also reflected in the low correlation of 0.27 (insert correlation) between ranking desert first in the ranking question and giving a response that can be coded as suffering in the open-ended question.

**Table 2:** Ranking aims of punishment: N and percentage across ranks.

	Amends	Deterrence	Disapproval	Reintegration	Desert
Rank1	55 (6.2)	301 (34.2)	81 (9.2)	235 (26.7)	209 (23.7)
Rank2	122 (13.8)	260 (29.5)	139 (15.8)	207 (23.5)	153 (17.4)
Rank3	202 (22.9)	193 (21.9)	164 (18.6)	170 (19.3)	152 (17.3)
Rank4	261 (29.6)	90 (10.2)	213 (24.2)	160 (18.2)	157 (17.8)
Rank5	241 (27.4)	37 (4.2)	284 (32.2)	109 (12.4)	210 (23.8)

**Fig 4:**
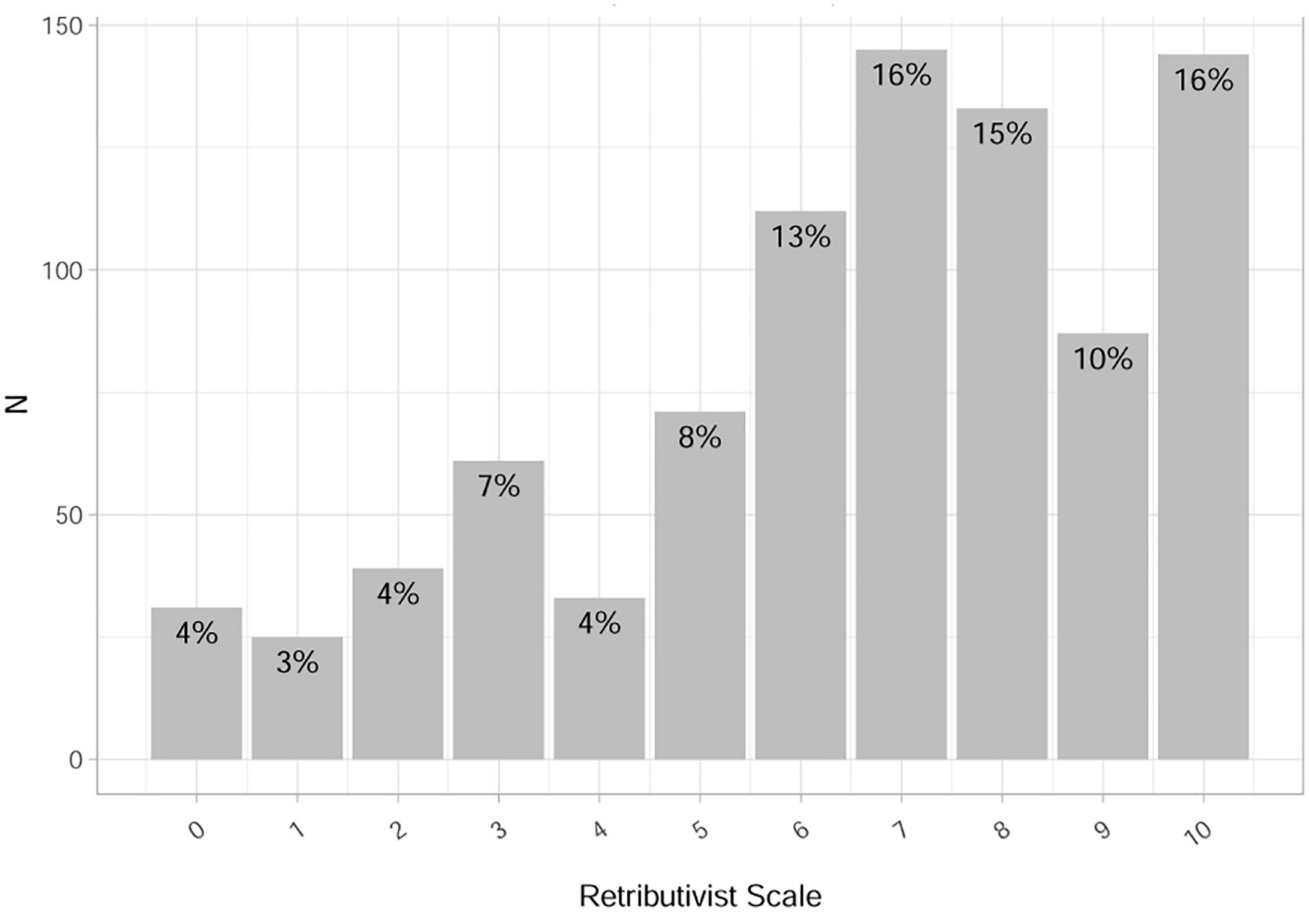
Retributivist scale: Distribution (N = 881).

The results for the third measure—the classically used scale—is depicted in Fig 4. While there is no plurality choosing suffering as an aim in our open-ended or our ranking question, 70% choose values above 5 on the classic scale. It is somewhat hard to translate the plurality hypothesis onto this scale, i.e., to pick a cut-off value above which we designate respondents as retributivists. Nonetheless, the fact that so many respondents locate themselves in the upper half of the scale (above 5) could be regarded as evidence supporting H1 and, thus, opposes evidence from the other two measures. In this sense, many respondents might be ‘basic retributivists’ [13].

Since, scholars do not seem to agree on which measure is more valid, we simply present evidence from all three measures regarding H1, i.e., that a plurality (the largest share) of people consider that the just response to serious criminal wrongdoing consists in making guilty offenders suffer for their crimes. Our findings serve as a reminder of how strongly it matters what measure we are using (S1 Appendix contains further analyses contrasting the three measures). Importantly, while the open-ended and the ranking measure provide evidence that goes against H1, they still indicate that retributive considerations do represent an important factor in assessing the morality of punishment. Respondents place these considerations before other considerations, such as making amends or disapproval by the society. Below we move on to our tests of Hypotheses 2 and 3.

### Hypothesis 2 and 3: Relevance of suffering and moral change

Fig 1 in the S1 Appendix visualizes the distribution of perceived justice, our outcome of interest for H2 and H3. Justice judgments are left-skewed, the mean lies at 5.94 and the standard deviation at 2.99.

**Fig 5:**
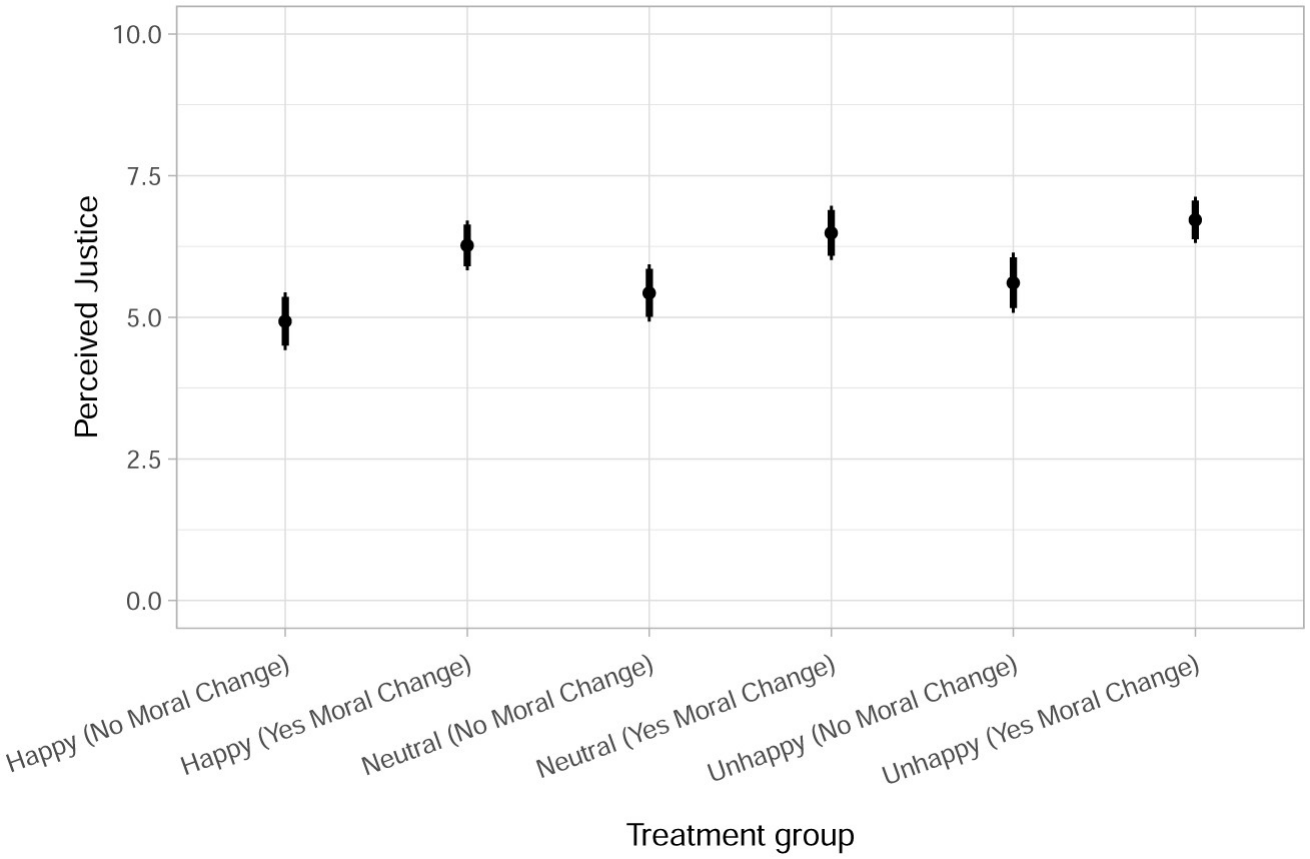
Perceived justice across treatment groups. Graph displays 95%- and 90%- confidence intervals as thinner and thicker bars; Data: original data.

Table 3 and Fig 5 provide mean comparisons, i.e., the average justice judgment across our different treatment groups. To assess the impact of our experimental treatment we start with some simple comparisons. Table 3 illustrates that means vary from a minimum of 4.93 to a maximum of 6.72, a difference of 1.79 points.

**Table 3:** Mean perceived justice across treatment levels.

Treatment group	Justice Perception (mean)	Justice Perception (sd)	N participants
Happy (No Moral Change)	4.93	3.15	147
Happy (Yes Moral Change)	6.27	2.73	150
Neutral (No Moral Change)	5.43	3.00	137
Neutral (Yes Moral Change)	6.49	2.86	138
Unhappy (No Moral Change)	5.61	3.13	133
Unhappy (Yes Moral Change)	6.72	2.75	176

Fig 5 visualizes the average on our justice judgments scale for the single treatment groups. There is a clear pattern for MoralChange. Individuals that receive the *yesmoralchange* treatment have higher levels of perceived justice than individuals that receive the *nomoralchange* treatment holding levels of suffering constant. At least visually the pattern is less clear for Suffering. It seems as if there is a slight increase in perceived justice with increased levels of suffering, namely moving from *happy* over *neutral* to *unhappy*. Albeit, visually, these differences are hardly distinguishable. For some basic ANOVA analyses we refer the reader to S1 Appendix.

In a next step we estimate a series of linear OLS regression models (see Table 4). Holding levels of moral change constant Suffering has a positive effect of 0.28 (Table 4, M3). This effect is statistically and also substantively significant: A move from Happy (Suffering variable = 0) to Unhappy (Suffering variable = 2) correspondends to a change of 0.56 on our outcome scale. Hence, we cannot reject Hypothesis 2 that *judgments of justice are positively affected by perceptions of the offender’s suffering*. This constitutes partial evidence in favor of the sufficiency claim. Holding levels of Suffering constant the effect of Moral Change on perceived justice is statistically significant and also substantively significant, namely 1.17 on an 11-point scale (Table 4, M3). Thus, we also find evidence in favour of Hypothesis 3, namely judgments of justice are positively affected by perceptions of the offender’s display of moral change. The effect of Moral Change on perceived justice seems substantively larger, however, this could also be due to the fact that the Moral Change manipulation takes up more room in our experimental treatments.

**Table 4:** Linear regression with and without manipulation checks.

	*Dependent variable*:					
	Perceived justice					
	M1	M2	M3	M4*	M5*	M6*
Moral Change (0,1)	1.20 *** (0.20)		1.17 *** (0.20)	1.23 *** (0.21)		1.20 *** (0.21)
Suffering (0-2)		0.32 ** (0.12)	0.28 * (0.12)		0.36 ** (0.13)	0.32 * (0.13)
Constant	5.31 *** (0.14)	5.62 *** (0.16)	5.04 *** (0.18)	5.36 *** (0.16)	5.65 *** (0.17)	5.05 *** (0.20)
Observations	881	881	881	772	772	772
R^2^	0.04	0.01	0.05	0.04	0.01	0.05

*p<0.05;

**p<0.01;

***p<0.001

* Models based on subset of participants that passed the manipulation check.

Models 4-6 reestimate Models 1-3 but are based on the subset of respondents that successfully answered the manipulation check questions. These were asked after our outcome measure and formulated as follows: “Please answer the following question: While in prison, David felt… very happy / neither happy nor unhappy / very unhappy / I don’t know” and “Please answer the following question: While in prison, David… wrote several apology letters to Julia / did not write any apology letter to Julia / I don’t know”. Respondents that provided the right answer to both, passed our manipulation check. The corresponding sample encompasses 772 instead of 881 respondents. As we would have expected the effects of our treatment are slightly stronger in this subsample, namely at 1.2 for Moral Change and 0.32 for Suffering.

## Discussion

The present study examines the evidence for two claims advanced by Michael Moore’s account of retributive justice. The first claim—the pervasiveness claim—suggests that people widely think that the just response to serious criminal wrongdoing is to make criminals suffer. Correspondingly, we hypothesized that a plurality (the largest group) of people thinks that the most important aim of punishment it to make guilty offenders suffer (H1). Evidence from the open-ended questions about the aims of punishment and from the ranking survey about the aims of punishment seems to speak against H1 and, consequently, seems to invalidate the pervasiveness claim. While there is a non-negligible proportion of respondents that mentions suffering in the open-ended questions, a plurality of people regards other aims of punishment as more important. Moreover, only 23.7% of the respondents ranked *giving offenders what they deserve* as the most important aim of punishment. Since it is plausible to assume that desert does not necessarily entail deserved suffering, this figure arguably offers an optimistic estimate of the pervasiveness of retributive beliefs.

Note, however, that 70% of the respondents chose values above 5 on the explicit retributivist scale, which was our third measure for the pervasiveness claim. This means that 70% of the respondents think that offenders deserve to suffer even if there are no other benefits of punishment for the offender or society as a whole. However, the size of these figures should be interpreted with a grain of salt. This is mainly because there is an ambiguity inbuilt in the explicit retributivist question, which asks “Do people who commit crimes deserve to suffer as a result of punishment, even if their suffering will not produce any positive benefits for the offender or society, like rehabilitation or the prevention of future crime?” The ambiguity comes from the fact that some people might have interpreted this question as asking whether, when punishment is *already* justified on some other ground than benefits like rehabilitation or prevention, punishment would still be justified as an imposition of deserved suffering on offenders (a better and less ambiguous question would have been to ask the following: “Is the fact that people who commit crimes deserve to suffer a sufficient reason to punish them, even if their suffering is the only effect of their punishment?”). This is a live possibility, given that, in their answers to the open-ended question about the aims of punishment, 23% of our respondents consider that making the offender aware of the harm or wrong done is the most important penal aim. If people have interpreted the explicit retributivist question in this way, then the corresponding retributivist scale provides a poor instrument for assessing the pervasiveness claim. This leads us to conclude that, based on the other two (less ambiguous) questions about the aims of punishment, evidence for the pervasiveness is questionable.

The second claim—the sufficiency claim—holds that suffering alone is sufficient to realize penal justice. We derived two hypotheses from this claim (one in agreement, one in disagreement with this claim): *judgments of justice are positively affected by perceptions of the offender’s suffering (H2)* and *judgments of justice are positively affected by an offender’s display of moral change (H3)*. We find that evidence on the sufficiency claim is mixed. Suffering has a positive effect on perceived justice (H2), which would speak in favor of the sufficiency claim. However, corresponding to H3, Moral Change has a stronger effect on perceived justice holding Suffering constant. Moreover, the levels of perceived justice in the moral change/suffering condition are higher than those in the no moral change/suffering condition. This suggests that perceived suffering does not, as such, provide sufficient grounds for evaluating the application of a criminal sanction as the best available realization of justice. Put differently, suffering does not suffice in those cases where participants’ judgments of justice register the highest level. Sure enough, neither does the offender’s moral change suffice for the application of a particular criminal sanction to be perceived as the best available realization of justice. But the sufficiency of moral change is not the normative claim on which Moore’s desert retributivism depends.

Positive retributivists might counter this interpretation by saying that, on reflection, people do not believe that moral change is *necessary* for realizing justice. Rather, moral change constitutes an additional consideration that would make punishment even more just than punishment limited to making guilty wrongdoers suffer. This rejoinder relies on a problematic premise. Suppose that we would have to choose between two instantiations of a given practice P—call these instantiations P1 and P2—each of which represents the realization of a justice ideal J by P. The perfect realization of J by P would count as 1, and the non-realization of J by P would count as 0. Now suppose that, assuming that a Pn which realizes J at 1 is virtually impossible—which makes sense if we think of just punishment as a procedurally imperfect form of justice, as theorists of justice tend to do [35]—and that P2 is the best realization of justice that is practically available, we have to choose between P1 where J = 0.6 and P2 where J = 0.7. Would we, in this situation, agree that P1 is sufficient for realizing J? We would not. This is because settling for anything less than the maximum satisfaction of J that is practically available constitutes an injustice, especially when the difference between the maximum and the next available option is substantial. Arguing that moral change is not necessary for just punishment to be realized is, we think, the same as arguing that it is acceptable to do *substantially* less than what we believe to be just when doing what we believe to be just is practically available to us. Ultimately, this is the same as saying that some degree of *injustice* is acceptable. Coming back to the sufficiency claim, it is the same as saying that some injustice is acceptable as long as no more than deserved suffering is realized through punishment. This hardly vindicates the claim that deserved suffering is sufficient for realizing justice through punishment.

One might alternatively contend that the realization of justice is not a matter of degree, but a categorical one: either justice is realized or it is not. But this cannot vindicate the sufficiency claim either. If nothing *else* than J = 1 constitutes justice, then, on people’s considered views about justice, neither suffering nor moral change (nor both) are sufficient for realizing justice. However, if J = n, where n is the best practically available approximation of J, then, in our case, people’s considered views are that moral change is necessary for n and, as a consequence, testify that suffering is not sufficient for the best available realization of justice.

One might, at a different level, worry that, in eliciting people’s judgments only from a third-personal perspective, we are neglecting Moore’s grounding of retributive judgments in a first-personal perspective. Moore holds that a first-personal perspective allows us to experience epistemically virtuous emotions such as guilt which, in turn, provide us with a reliable way of grasping the truth of retributive justice [3]. The worry, then, would be that, by concentrating on judgments of justice considered from a third-personal perspective (as judgments about David Chadwick), we are failing to consider judgments cast from a first-personal perspective (where respondents would have to imagine that they themselves are David Chadwick) which might be more markedly retributive in Moore’s sense. There are at least three reasons to think that this worry is overstated. First, Moore never argues that the first-personal perspective is in itself normatively prior to the third-personal one. The third-personal emotion of moral outrage one experiences at another person’s crimes is not normatively less weighty than the first-personal emotion of guilt that one would experience at one’s own crimes. It thus seems safe to assume that both perspectives and their corresponding retributive emotions (moral hatred and guilt) have roughly equivalent evidential credentials [3].

Second, Moore’s move from the third-personal perspective to the first-personal one is explicitly presented as an attempt to show that retributive judgments are not reducible to normatively problematic expressions of third-personal ressentiment [3]. But if ressentiment makes people more retributive than they should otherwise be, our focus on the third-personal perspective most likely offers an overestimate of the weight that respondents give to deserved suffering in their considered judgments of justice. As such, our findings could not be unduly biased against desert retributivism.

Third, in the writing-up the vignette, we were careful not to include any compassion-eliciting fact or event that could have rendered respondents less responsive to retributive considerations. In so doing, the deck is not stacked against retributive emotions such as moral outrage. More generally, asking people to imagine that they committed a rape raises intricate ethical issues that we thought should be avoided and could be avoided at no considerable theoretical cost by privileging the third-personal perspective over the first-personal one.

One might also argue that people’s views about just punishment do not have the same evidential weight as philosophical views and intuitions about justice. This is an uncomfortable move to make for Moore or for anyone who is committed to a reflective equilibrium approach to justification. On such an approach, people’s well-considered judgments and views about justice have a *prima facie* equal evidential weight when it comes to deciding what constitutes justice [36]. Since we do not have any good reasons to think that the justice views and intuitions of our respondents were systematically distorted by morally problematic factors and since there is evidence that philosophers’ substantive intuitions about justice are not systematically better than ordinary people’s views and intuitions [37, 38], we do not have any good reasons to discredit the evidential weight of ordinary views and intuitions about justice. It would nonetheless be useful to see if justice views and intuitions vary according to specific demographics such as profession where lawyers or philosophers might systematically hold different views of justice or education.

This brings us to conclude that the evidence in favor of the sufficiency claim is, under the most charitable interpretation, mixed. Perceived suffering is positively correlated with people’s judgments of justice. This satisfies the first dimension of sufficiency relations that we formulated in Section 2, namely the positive valence of the relation between the sufficiency condition and the thing that condition is supposedly sufficient for (in our case, justice judgments about punishment). However, moral change seems necessary for people to perceive punishment as more fully just. This testifies to the absence of the non-necessity of alternate conditions dimension of sufficiency conditions, where no condition other than the sufficiency condition is necessary for the thing that condition is supposedly sufficient for.

## Conclusion

This study offers the first experimental assessment of positive retributivism, which argues that the deserved suffering of offenders provides a sufficient reason for punishing them. Our findings suggest that people do not pervasively support positive retributivism, and provide mixed evidence that deserved suffering is sufficient for just punishment.

Our study is limited in various ways. These may serve as starting points for future research. First, our findings are based on an online survey. This does not call into question the experimental results since we randomize respondents into conditions. However, it may affect the extent to which one can generalize from our findings to other populations, as online respondents are not representative of the wider population. While our sample is diverse, i.e., more representative than a student sample, future studies should try to collect data that is randomly sampled from registers such as is regularly done in bigger survey projects. Second, our study is limited to only one offense category (rape). Even if, given the seriousness of rape, our study suggests that deserved suffering might not be widely considered as a sufficient penal justification in other (less serious) cases, a full assessment of positive retributivism would need to examine the role that deserved suffering plays in the case of other offense categories, such as murder or property crimes. Third, and finally, future studies should explore other possible operationalizations of deserved suffering—for instance, *painful* or *sad*—that would, when considered in conjunction with our own operationalization (*unhappy*), more closely capture the moral complexity of the phenomenon of suffering. Additionally, one should validate that offenders described with such states are really perceived as suffering by experimental subjects, e.g., by letting a subsample of the participants rate how they perceived an offender’s suffering.

Before concluding, we would like to consider another objection. One might argue that our mixed findings about retributive judgments in Moore’s sense offer are limited, and that more evidence is needed before casting doubt on desert retributivism. This is a sensible suggestion, but cannot vindicate desert retributivism as we’ve construed it. There is indeed some evidence that, on the whole, retributivism tends to be endorsed by ordinary people in specific cases of criminal wrongdoing. For instance, [39] find that desert considerations—in particular, the seriousness of a criminal wrong—and not incapacitation ones are central to explaining both the severity of punishment (i.e. the extent to which offenders should be punished) and the length of criminal sentences. Similarly, [40] find that people tend to impose longer sentences when wrongs are intentional (as crimes are generally considered to be), and take a principle of just deserts to account for this tendency. [41] and [42] concludes that concrete sentencing decisions about how much any individual offender should be punished are retributive, in the sense that they generally track the magnitude of the criminal wrong, and not their preventive potential. This is so despite people’s verbally professed attachment to deterrence as their preferred penal justification. Finally, [43] argues that, since people tend to overwhelmingly agree that the offense seriousness is decisive for punishment severity, we should accept the principle of just deserts as a universal justificatory basis for punishment.

While informative, these studies cannot conclusively support desert retributivism as we’ve construed it. There are two main reasons for this. First, they tend to operate with a strictly quantitative dependent variable, which is sentence length. While sentence length might be a plausible proxy for deserved penal suffering, it might also differ from it, especially when differences in sentence length are not substantial. Most notably, sentence length or proportionality between offense seriousness and sentence length might be driven by non-retributive confounds that are hard to detect given these studies’ design. Second, there is counter-evidence that ordinary people don’t take suffering as such to justify punishment, even when suffering is deserved. For instance, [44] show that the harm caused by penal sanctions in a laboratory setting does not *as such* satisfy punishers and that, on the contrary, punishers tend to feel worse rather than better about their penal decisions once they impose them. Similarly, [45] and [46] find that people don’t consider suffering to intrinsically justify punishment, but rather tend to take suffering as an instrumentally valuable medium that shows whether offenders understood their offense was morally wrong, and thus conclude that “desires to worsen the offender’s emotional state might simply be a proxy for more functional latent goals—such as delivering a message to the offender” [46]. Finally, [47]’s in-lab experimental studies provide evidence that participants who were allowed to sanction specific transgressions experienced significant justice-related satisfaction only when transgressors showed remorse for their transgression, but not when they merely suffered for it. This suggests, once again, that suffering is instrumentally valuable as a mechanism for communicating a moral message, but not intrinsically valuable, as desert retributivists claim it is. For more general theoretical discussion of these and other studies, see [12].

To this, one might reply that desert retributivism might be pervasively present in a different way or, better put, at a different level. Innovative studies [13, 48] offer some evidence that many (though not most) people prefer to engage in punishing those who violate generally accepted fairness norms, even when punishment is resource-costly and remains secret such that it cannot function as a moral communication device. This “hidden punishment” [48] phenomenon has prompted some philosophers to argue that imposing some suffering on wrongdoers even if doing so serves no other moral purpose might simply be “a basic, independent part of our moral worldview” [49]. On this interpretation, we are all pervasively, if hiddenly, retributivists. But even if basic retributivism might partly support the retributivist’s pervasiveness claim, it cannot support its sufficiency claim. Largely unreflective norms cannot in and of themselves provide enough reason to punish, nor can they provide conclusive reasons to punish for most of us. This might explain why, in the study by [48], costly punishment rates were significantly higher when punishment was *not* hidden. Note also that basic retributivism is not the only way one might interpret the hidden punishment phenomenon. Drawing on [50], one might alternatively contend that hidden punishment is prompted by a heuristic whereby people continue to punish because they (inertly) care about their reputation and trustworthiness even when their acts of costly punishment are unlikely to be observed by others.

Looking beyond our study to other experimental studies does not offer a conclusive case for desert retributivism. Instead, it only shows that desert retributivism—i.e. the theory that takes deserved suffering to intrinsically justify punishment—is supported by some of our ordinary views about justified punishment, and that some of these retributive views are unreflective, unconscious and automatic. Given Moore’s and other desert retributivists’ commitment to reflective equilibrium, this might well mean that deserved suffering cannot ultimately count as a reflectively held justification for punishment. Put differently, retributive justice seems to be caught in an ongoing process of reflective *disequilibrium* where it persistently and unresolvedly competes with other justificatory principles like deterrence or the offender’s moral reform. Note also that the limited to null support that desert retributivism obtains in the Chadwick case we study is especially relevant. The vignette portrays a central, because particularly grave case of moral wrongdoing (rape). Given their subject-matter, judgments about the Chadwick case could be considered as prima facie weightier than judgments about other, less wrong, criminal cases. If retributive judgments are at their maximum in the Chadwick case, it might turn out that desert retributivism cannot hope for much normative support when considering other, less grave cases of criminal wrongdoing.

## Supporting information

S1 Appendix (PDF)
