# Supplementary material for: Does suffering suffice? An experimental assessment of desert retributivism
Source: PLoS One. 2020 Apr 20;15(4):e0230304. doi: 10.1371/journal.pone.0230304 (PMC7170504; doi:10.1371/journal.pone.0230304)
Supplement: S1 Appendix — (PDF) [file pone.0230304.s001.pdf]

# S1 Appendix: Does Suffering Suffice? An Experimental Assessment of Desert Retributivism

# Contents

**Summary statistics** **3**

**Balance statistics** **3**

**Perceived justice: Distribution** **4**

**Crowd-coding of open-ended responses** **4**

**Analysis of variance** **7**

**Contrasting open-ended, ranking and classic retributivism scale** **9**

**R session info** **11**

**References** **12**

## Summary statistics

Table A displays summary statistics for the numeric variables. For the categorical variables we refer to the graphs in the manuscript.

Table A: Summary stats for age and sex

| Statistic | N | Mean | St. Dev. | Min | Pctl(25) | Pctl(75) | Max |
|---|---|---|---|---|---|---|---|
| age | 881 | 38.90 | 12.04 | 18 | 30 | 47 | 73 |
| sex | 881 | 0.50 | 0.50 | 0 | 0 | 1 | 1 |

## Balance statistics

Table B provides balance statistics for sex and age.

Table B: Balance Statistics: Sex, Age

| | Sex (Mean) | Age (Mean) | N (total) |
|---|---|---|---|
| Happy (No Moral Change) | 0.50 | 39.59 | 147 |
| Happy (Yes Moral Change) | 0.53 | 38.98 | 150 |
| Neutral (No Moral Change) | 0.48 | 37.80 | 137 |
| Neutral (Yes Moral Change) | 0.54 | 38.65 | 138 |
| Unhappy (No Moral Change) | 0.48 | 39.55 | 133 |
| Unhappy (Yes Moral Change) | 0.46 | 38.84 | 176 |

# Perceived justice: Distribution

Fig A: Perceived justice: Distribution

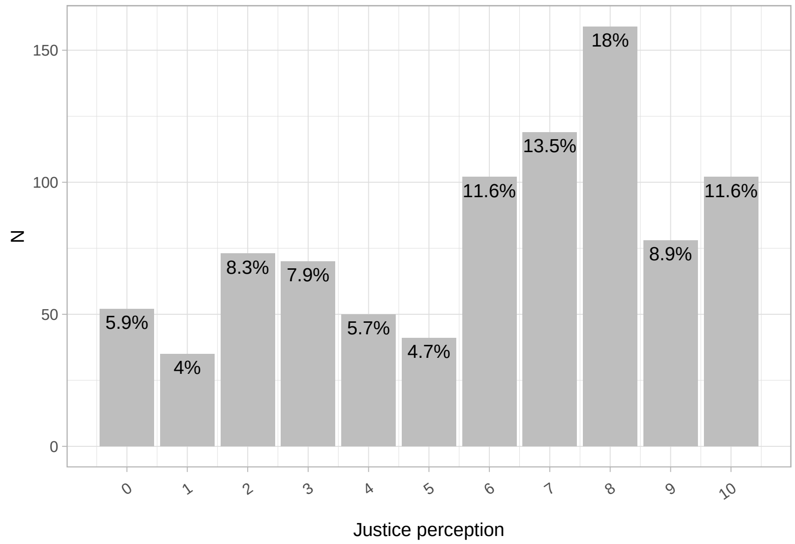


# Crowd-coding of open-ended responses

In total 119 mechanical turk workers participated in our crowd-sourcing task to classify responses to our open-ended question on aims of punishment. Table C provides some statistics on the crowdsourcing task. We had 881 responses. The idea was to classify each response by 4 raters which would result in a total number of 3524 assignments. In the end our data comprised 3466 analyzable assignments. We crowd-sourced the data in 5 batches in order to be able to assess the rating quality and other statistics along the way. As suggested by [1] we tried to pay workers above the minimum wage of 7.25$. On average our workers recieved a wage of 7.42 $ per hour. Depending on their speed their wage may vary. Mechanical turk workers that were accepted for our task needed to be located in the U.S., have a HIT Approval Rate (%) for all Requesters' HITs greater than 97%, have a number of HITs Approved greater than 1000 and needed to have 'Masters' granted. Masters are elite groups of Workers who have demonstrated accuracy on specific types of HITs on the Mechanical Turk marketplace. We added Masters requirement after Batch 1 and noticed a considerable increase in response quality.
The crowd-sourcing task is depicted in Figure B. We provided raters with a set of possible aims of punishment and asked them to classify the responses regarding whether certain aims were mentioned or implied by a respondent's answer. For this task we did not randomize the ranking of the categories since we wanted raters to get used to the classification interface.

Since not all raters coded all responses we use Krippendorf's alpha as a measure of interrater reliability [2]. We calculated alpha for each of the 7 categories into which raters could categorize a response. The results are depicted in Table D. Krippendorf's Alpha ranges from 0.33 to 0.74, i.e., we get categories for which it is relatively satisfying, e.g., rehabilitation, and categories for which is less satisfying, e.g., vengeance.

Table C: Crowdsourcing stats

| Statistic | Value |
|---|---|
| Time minimum (minutes) | 0.03 |
| Time maximum (minutes) | 5.93 |
| Average time per assignment (minutes) | 1.65 |
| Total time (minutes) | 5731.23 |
| Total time (hours) | 95.52 |
| Average pay per assignment (cent) | 20.46 |

Table D: Interrater-reliability: Krippendorf's alpha

| Category | Alpha | Responses | Raters |
|---|---|---|---|
| Suffering | 0.49 | 881 | 119 |
| Deterrence | 0.64 | 881 | 119 |
| Reintegration | 0.36 | 881 | 119 |
| Rehabilitation | 0.74 | 881 | 119 |
| Amends | 0.38 | 881 | 119 |
| Vengeance | 0.33 | 881 | 119 |
| Awareness | 0.63 | 881 | 119 |

Fig B: Crowd-coding of responses: Aims of punishment

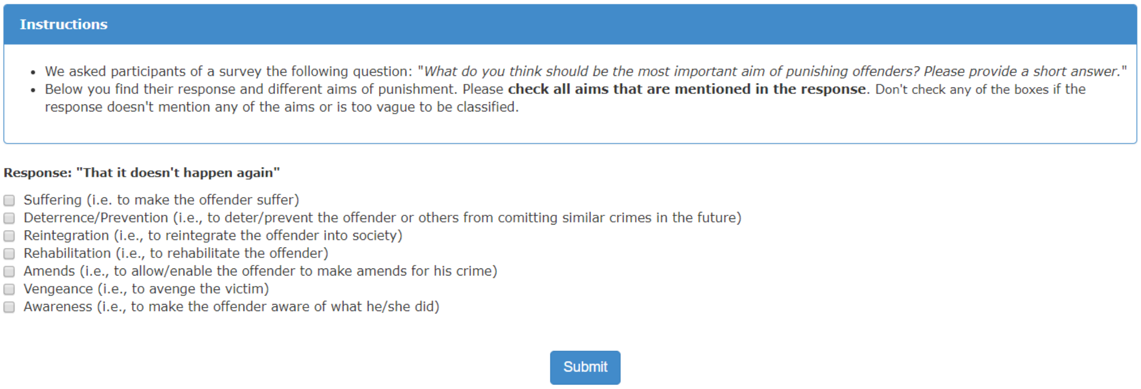

**Instructions**

- We asked participants of a survey the following question: "*What do you think should be the most important aim of punishing offenders? Please provide a short answer.*"
- Below you find their response and different aims of punishment. Please **check all aims that are mentioned in the response**. Don't check any of the boxes if the response doesn't mention any of the aims or is too vague to be classified.

**Response: "That it doesn't happen again"**

- Suffering (i.e. to make the offender suffer)
- Deterrence/Prevention (i.e., to deter/prevent the offender or others from comitting similar crimes in the future)
- Reintegration (i.e., to reintegrate the offender into society)
- Rehabilitation (i.e., to rehabilitate the offender)
- Amends (i.e., to allow/enable the offender to make amends for his crime)
- Vengeance (i.e., to avenge the victim)
- Awareness (i.e., to make the offender aware of what he/she did)

Submit

For the main analysis in the paper we chose a conservative strategy. We only coded a response as belonging to a category such as "suffering" when at least 3 out of 4 raters agreed that it belonged to that particular category. This is a rather strict cutoff and could mean that we underestimate the prevalence of certain aims in the responses. However, we assume that any such underestimation is relatively constant across aims, hence, it shouldn't affect our conclusions about Hypothesis 1. Crowd-coding is both hailed as a useful strategy but also viewed critically [3–6]. Because Krippendorf's alpha was not higher for certain categories we carried out additonal analyses to see whether our results remain robust to the exclusion of certain workers. Some workers may take the task less seriously than others which leads to measurement error. Below we excluded the codings of workers that finished the assignments in an average time lower than 0.3 minutes, or longer than 5 minutes as well as coded only 1 response. Extremely low average times may reflect superficial codings. Very long times may indicate that workers worked on several parallel assignments and only finished them once the time ran out. Furthermore, we assume that the quality of coding may improve once workers get used to the coding scheme. The results for this rater subsample are depicted in Table E and

Table E: Interrater-reliability: Krippendorf's alpha

| Category | Alpha | Responses | Raters |
|---|---|---|---|
| Suffering | 0.49 | 881 | 67 |
| Deterrence | 0.67 | 881 | 67 |
| Reintegration | 0.36 | 881 | 67 |
| Rehabilitation | 0.76 | 881 | 67 |
| Amends | 0.42 | 881 | 67 |
| Vengeance | 0.39 | 881 | 67 |
| Awareness | 0.65 | 881 | 67 |

Table F: Share of open-ended answers that mention particular aims

| | % responses | N responses |
|---|---|---|
| Mention aim of suffering | 7 | 63 |
| Mention aim of deterrence | 24 | 212 |
| Mention aim of reintegration | 1 | 13 |
| Mention aim of rehabilitation | 24 | 208 |
| Mention aim of amends | 3 | 27 |
| Mention aim of vengeance | 2 | 20 |
| Mention aim of awareness | 15 | 135 |

Table F. Krippendorf's alpha slightly increase for most of the categories. However, the main findings, namely the comparably low share of responses mentioning suffering as aim of punishment, does not change. In Table F the share of responses that are classified as mentioning the aim of suffering is even lower than before the exclusion of certain raters.

Finally, while Table F depicts the prevalence of certain aims across all respondents, Table G depicts the prevalence of certain aims of punishment split across treatment groups. In other words, since we collected the data to test H1 after our survey experiment we could be worried that the considerations queried through the open-ended question are affected by our survey experiment. Table G allows us to explore whether participants's open-ended answers seem to have been influenced by our experimental treatments, i.e., by our experiment. While there are some differences these do not seem to be strong enough to be problematic for a test of Hypothesis 1.

Table G: Share of open-ended responses that mention particular justifications/aims across treatment groups

| Treatment | Mention suffering (%) | Mention deterrence (%) | Mention reintegration (%) | Mention rehabilitation (%) | Mention amends (%) | Mention vengeance (%) | Mention awareness (%) |
|---|---|---|---|---|---|---|---|
| happy__nomoralch | 8 | 20 | 1 | 22 | 3 | 1 | 18 |
| happy__yesmoralch | 7 | 25 | 1 | 22 | 3 | 2 | 9 |
| neutral__nomoralch | 8 | 20 | 1 | 19 | 2 | 1 | 17 |
| neutral__yesmoralch | 6 | 23 | 1 | 26 | 4 | 7 | 16 |
| unhappy__nomoralch | 5 | 22 | 0 | 27 | 5 | 1 | 19 |
| unhappy__yesmoralch | 8 | 32 | 4 | 25 | 2 | 2 | 14 |

## Analysis of variance

In addition to the comparisons and models estimated in our 'Results' Section we carried out classical ANOVA analyses. Figure C displays the averages across all treatment groups. Figure D displays the averages in the treatment groups with samples being split according to values of our two treatment variables — Suffering and Moral Change — independently from the respective other variable. The actual data was spread out using jitter.

Fig C: Means and distributions across all treatment groups

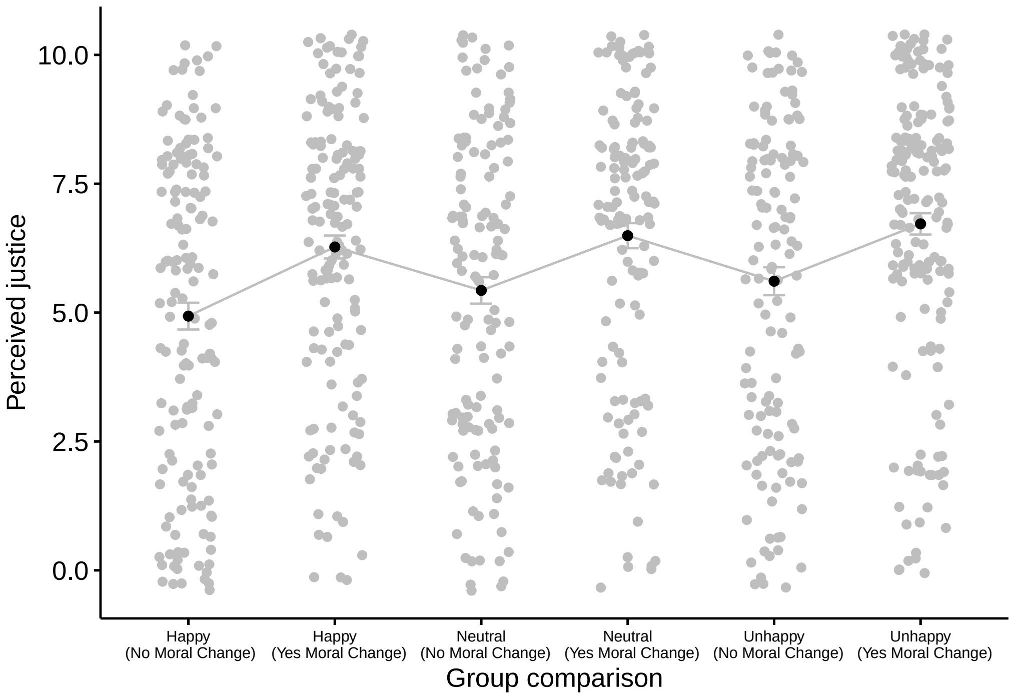


One-way ANOVA tests yield significant p-values for groups means for both the Suffering treatment (P-value = 0.033) and Moral Change treatment (P-value = 2.15e-09) indicating that some of the group means are different. While there are only two subsamples (groups or values) for Moral Change, we don't know which combinations of the three Suffering subsamples (groups or values) display statistically significant differences. One-way ANOVA tests splitting the sample into groups corresponding to the 6 treatment groups yield the same result.

In a next step we perform multiple pairwise-comparison computing Tukey Honest Significant Differences [7], to determine if the mean difference between specific pairs of groups are statistically significant. We find that there is a highly statistically significant difference comparing the Moral Change treatments ("no" vs. "yes"). The difference lies at 1.2 (P-value = 0.00). For Suffering there is a significant difference of -0.63 when we compare the "unhappy" to the "happy" category (P-value = 0.02), i.e., the two extreme categories on this three-point scale. The differences between neutral-unhappy and happy-neutral are not statistically significant. ANOVA tests assume normally distributed data and homogeneous variance across groups. We checked the homogeneity of variance assumption relying on Levene's test [8]. The test indicates a violation for groups of Moral Change but not for groups of Suffering. For this reason we compute a non-parametric alternative to the one-way ANOVA test, namely the Kruskal-Wallis rank sum test [9]. The results from the rank sum test indicate that there are significant differences between our treatment groups for our two

Fig D: Means and distributions for sample split according to the two treatment variables

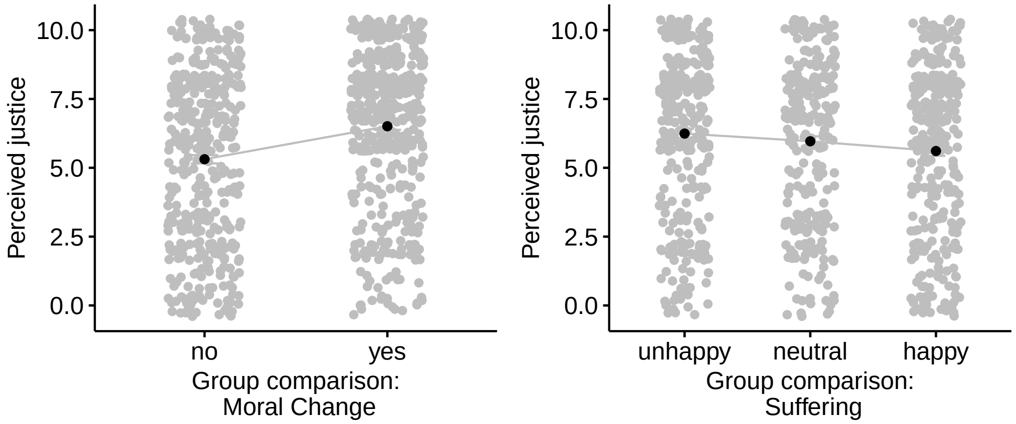


treatment variables Moral Change and Suffering. These results reflect the findings from our main analysis. For this reason we refer the reader back to the 'Results' Section in the main paper.

# Contrasting open-ended, ranking and classic retributivism scale

Table H displays the open-ended responses after they have been classified according to whether they mentioned particular aims of punishment. However, as opposed to Table 1 in the main paper we now show the marginal distributions for different values on the retributivism scale that has 11 values. Specifically, we show those distributions for respondents with low values on the scale (0-3) and for respondents with high values on the scale (7-10). As was to be expected the share of respondents that mention suffering as an aim in their open-ended response is higher among those that also picked high values on the retributivsm scale. Nontheless, those shares are lower than one would expect. To some extent this is certainly related to the way we coded those open-ended responses. However, even if those values would vary because of a different coding scheme, the numbers would still be in the lower range. Further below we contrast the explicit retributivism scale with the ranking question on aims of punishment.

Table H: Share of open-ended answers that mention particular aims for particular reponses on the closed retributivist scale

| | 0 | 1 | 2 | 3 | 7 | 8 | 9 | 10 |
|---|---|---|---|---|---|---|---|---|
| Mention aim of suffering | 0 (0) | 0 (0) | 0 (0) | 0 (0) | 0.07 (10) | 0.07 (9) | 0.17 (15) | 0.14 (20) |
| Mention aim of deterrence | 0.16 (5) | 0.12 (3) | 0.28 (11) | 0.26 (16) | 0.26 (38) | 0.23 (31) | 0.25 (22) | 0.24 (35) |
| Mention aim of reintegration | 0 (0) | 0 (0) | 0.05 (2) | 0.03 (2) | 0.01 (1) | 0.02 (3) | 0.01 (1) | 0 (0) |
| Mention aim of rehabilitation | 0.39 (12) | 0.32 (8) | 0.31 (12) | 0.33 (20) | 0.24 (35) | 0.17 (23) | 0.17 (15) | 0.12 (18) |
| Mention aim of amends | 0.03 (1) | 0.08 (2) | 0 (0) | 0.03 (2) | 0.03 (5) | 0.02 (3) | 0 (0) | 0.01 (2) |
| Mention aim of vengeance | 0.03 (1) | 0.08 (2) | 0.03 (1) | 0 (0) | 0.03 (5) | 0.02 (3) | 0.05 (4) | 0.01 (1) |
| Mention aim of awareness | 0.1 (3) | 0.08 (2) | 0.15 (6) | 0.2 (12) | 0.15 (22) | 0.22 (29) | 0.11 (10) | 0.12 (17) |

Figure E visualizes the results of the ranking question that provides respondents with a pre-defined choice set of aims of punishment. However, now we visualize those rankings for subsets of participants that picked particular values on the retributivism scale, either low values (0-3) or high values (7-10). Again we can observe that respondents that pick high values on the retributivism scale more often rank the aim of desert first. However, by far not everyone does. For instance, across both low and high values of the classic retributivism scale a large share of people rank the aim of deterrence in the first place. In other words, when contrasted with the classic retributivism scale both our open-ended measure and our ranking measure reveal that while there is overlap, there is also considerable variation behind the same value on this scale.

Fig E: Rankings of aims for different values on the redistributive scale

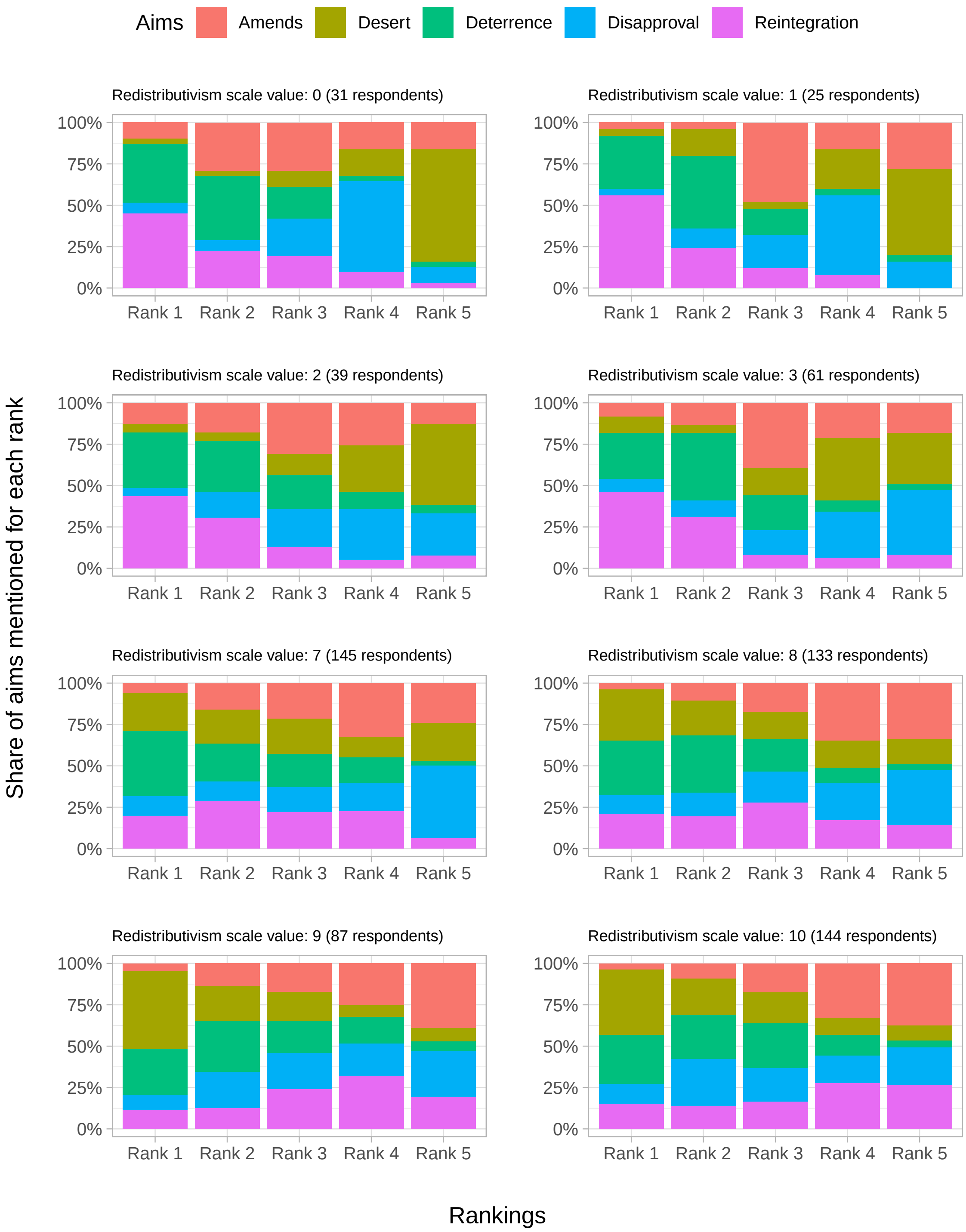

# R session info

```
## R version 3.6.2 (2019-12-12)
## Platform: x86_64-w64-mingw32/x64 (64-bit)
## Running under: Windows 10 x64 (build 17763)
##
## Matrix products: default
##
## attached base packages:
## [1] grid      stats     graphics  grDevices utils     datasets  methods
## [8] base
##
## other attached packages:
##  [1] purrr_0.3.3      gridExtra_2.3    ggpubr_0.2.4     magrittr_1.5
##  [5] irr_0.84.1       lpSolve_5.6.13.3 tidyr_1.0.0      kableExtra_1.1.0
##  [9] xtable_1.8-4     stringr_1.4.0    readr_1.3.1      stargazer_5.2.2
## [13] dplyr_0.8.3      plotly_4.9.1     ggplot2_3.2.1    haven_2.2.0
## [17] knitr_1.26
##
## loaded via a namespace (and not attached):
##  [1] tidyselect_0.2.5  xfun_0.11         colorspace_1.4-1  vctrs_0.2.4
##  [5] htmltools_0.4.0   viridisLite_0.3.0 yaml_2.2.0        rlang_0.4.5
##  [9] pillar_1.4.3      glue_1.3.2        withr_2.1.2       lifecycle_0.2.0
## [13] munsell_0.5.0     ggsignif_0.6.0    gtable_0.3.0      rvest_0.3.5
## [17] htmlwidgets_1.5.1 evaluate_0.14     labeling_0.3      forcats_0.4.0
## [21] Rcpp_1.0.3        scales_1.1.0      webshot_0.5.2     jsonlite_1.6
## [25] farver_2.0.1      hms_0.5.2         digest_0.6.23     stringi_1.4.3
## [29] bookdown_0.16     tools_3.6.2       lazyeval_0.2.2    tibble_2.1.3
## [33] crayon_1.3.4      pkgconfig_2.0.3   ellipsis_0.3.0    data.table_1.12.8
## [37] xml2_1.2.2        assertthat_0.2.1  rmarkdown_2.0     httr_1.4.1
## [41] rstudioapi_0.10   R6_2.4.1          compiler_3.6.2
```

# References


1. Williamson V. On the ethics of crowdsourced research. PS Polit Sci Polit. Cambridge University Press; 2016;49: 77–81.

2. Krippendorff K. Content analysis: An introduction to its methodology. Thousand Oaks, CA: Sage; 2013.

3. Snow R, O'Connor B, Jurafsky D, Ng AY. Cheap and fast—but is it good?: Evaluating non-expert annotations for natural language tasks. Proceedings of the conference on empirical methods in natural language processing. Stroudsburg, PA, USA: Association for Computational Linguistics; 2008. pp. 254–263.

4. Benoit K, Conway D, Lauderdale BE, Laver M, Mikhaylov S. Crowd-sourced text analysis: Reproducible and agile production of political data. Am Polit Sci Rev. Cambridge University Press; 2016;110: 278–295.

5. Lind F, Gruber M, Boomgaarden HG. Content analysis by the crowd: Assessing the usability of crowdsourcing for coding latent constructs. Commun Methods Meas. 2017;11: 191–209.

6. Dreyfuss E, Barrett B, Newman LH. A bot panic hits amazon's mechanical turk. Wired. WIRED; 2018;

7. Yandell B. Practical data analysis for designed experiments. Routledge; 2017.

8. Fox J. Applied regression analysis and generalized linear models. Sage; 2016.

9. Hollander M, Wolfe DA. Nonparametric statistical methods. John Wiley & Sons; 1973.
